# Precise reconstruction of the aortic valve using glutaraldehyde-treated autologous pericardium in new formula: a case report

**DOI:** 10.1186/s13256-024-04637-6

**Published:** 2024-07-09

**Authors:** Feridoun Sabzi, Aghigh Heydari, Atefeh Asadmobini

**Affiliations:** Cardiovascular Research Center, Kermanshah University of Medical Sciences, Kermanshah, Iran

**Keywords:** Aortic valve reconstruction, Glutaraldehyde-treated autologous pericardium, Case report

## Abstract

**Background:**

The current study presents a novel and precise surgical technique for complete reconstruction of the aortic valve using glutaraldehyde-treated autologous pericardium in a patient with aortic valve disease and endocarditis. The technique aims to provide a more effective and reproducible method for aortic valve repair, with the goal of improving outcomes and quality of life for patients with aortic valve disease.

**Case presentation:**

A 35-year-old Iranian male with aortic valve disease and endocarditis underwent aortic valve reconstruction surgery. Preoperative echocardiography showed a degenerative aortic valve with severe regurgitation, reduced left ventricular ejection fraction, and specific aortic root dimensions. The surgical technique involved precise measurements and calculations to design the size and shape of the new aortic valve cusps using autologous pericardium, with the goal of optimizing coaptation and function. The surgeon calculated the intercommissural distance based on the aortic annulus diameter to determine cusp size and shape. He tailored the pericardial cusps to have a height equal to 80% of the coaptation margin length. Detailed suturing techniques were used to ensure proper alignment and coaptation of the new cusps. Intraoperative evaluation of the valve function using suction and transesophageal echocardiography showed good coaptation and minimal residual regurgitation. At the 3-year follow-up, the patient had a well-functioning aortic valve with only trivial leak and was in satisfactory clinical condition.

**Conclusions:**

Glutaraldehyde-treated autologous pericardium is a validated leaflet alternative, and the causes of its failure are late annular dilatation and other technique breakdowns. Current evidence reveals that aortic valve reconstruction with glutaraldehyde-treated autologous pericardium is associated with many advantages with the potential to improve patient outcomes and quality of life. Further clinical studies are warranted to evaluate the long-term durability and efficacy of this approach.

## Background

Aortic valve disease is a serious condition that affects the valve responsible for regulating blood flow from the heart to the aorta. This valve can become narrowed (stenosis) or leaky (regurgitation), leading to complications such as heart failure if left untreated [1]. Surgical repair or replacement of the aortic valve is often necessary to restore proper heart function and prevent life-threatening complications [2]. Traditional open-heart surgery has been the standard approach, but minimally invasive techniques such as mini-thoracotomy and robotic-assisted procedures have also been developed to reduce surgical trauma [3]. While significant progress has been made in aortic valve surgery, there is still a need to improve outcomes and provide more durable treatment options, especially for younger patients. Biological valve replacements have limitations such as finite lifespan, while mechanical valves require lifelong anticoagulation therapy [3]. Total aortic reconstruction by glutaraldehyde-treated autologous pericardium (GTAP) has been popularized by Duran and Ozaki in the last decades [4, 5]. Innovative surgical techniques like this are crucial, as aortic valve disease accounts for over 60% of all deaths from valve disease [6].

In the current study, a new technique for aortic valve reconstruction is presented, which uses a new formula to make the technique precise and reproducible. This approach aims to provide a more effective and reproducible method for aortic valve repair, with the goal of improving outcomes and quality of life for patients with aortic valve disease.

## Case presentation

### Patient history and preoperative evaluation

**Fig. 1:**
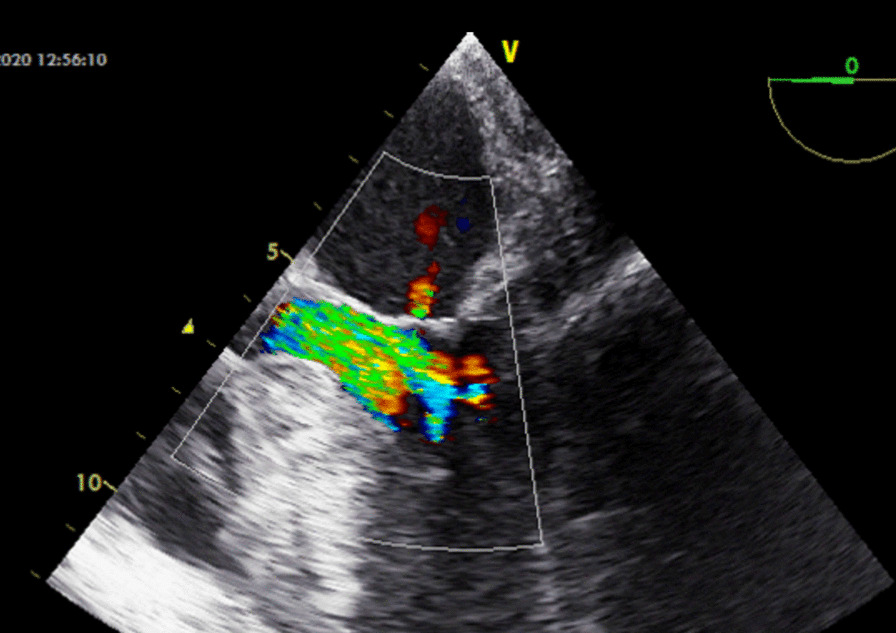
TEE long-axis view shows severe aortic regurgitation

**Fig. 2:**
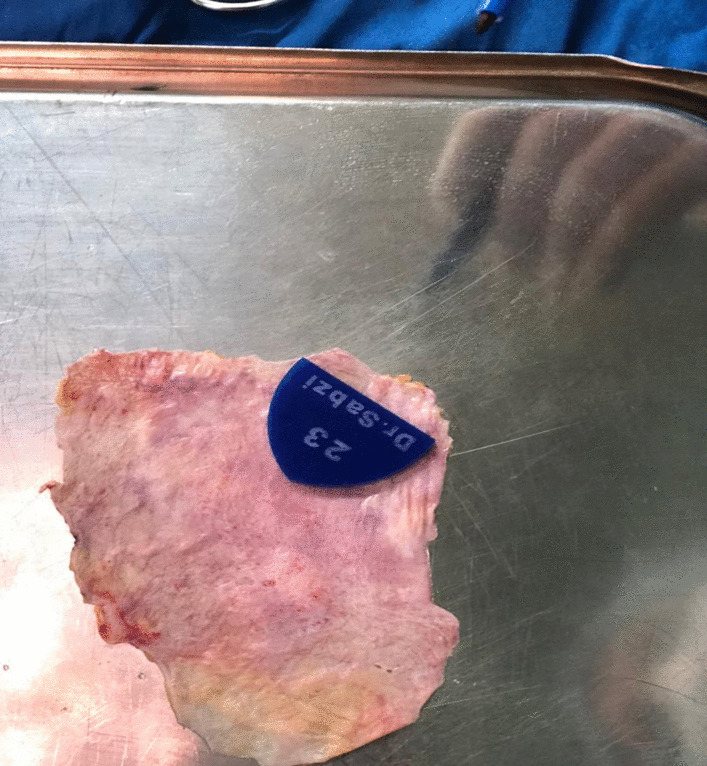
Constructed template by compacted plastic for each size of aortic ring diameter

A 35-year-old Iranian male patient was referred to the hospital with dyspnea due to aortic valve disease and endocarditis. Prior to surgery, the patient completed a 3-week course of antibiotic therapy and provided consent for the aortic valve reconstruction procedure. Preoperative transthoracic echocardiography (TTE) revealed a degenerative aortic valve with severe aortic regurgitation (Fig. 1). The TTE also showed a reduced left ventricular ejection fraction of 40% and an enlarged left ventricular end-systolic diameter of 54 mm. Measurements of the aortic root indicated an aortic annulus of 23 mm, sinotubular junction of 28 mm, and ascending aorta of 30 mm. The vena contracta of the aortic regurgitation was 7 mm. Anatomically, the size of the coaptation margin was 1.5 times larger than the aortic annulus margin. Therefore, in this article, to increase coaptation’s length, the measured size of coapting margin is considered as 1.8 times of intercommissural distance (ICD). By calculation of ICD and its multiplying by 1.8, marginal coaptation length is obtained. The size of new cusp radius is similar to ICD. As shown in Fig. 2 to design a leaflet, the height and coapting margin of the leaflet were marked on the pericardium. Then, a semicircle was drawn along the cusp’s height line (semicircle’s radius is equal to ICD), and the end of the semicircle was connected in a straight line to cross the coapting margin line.

**Fig. 3:**
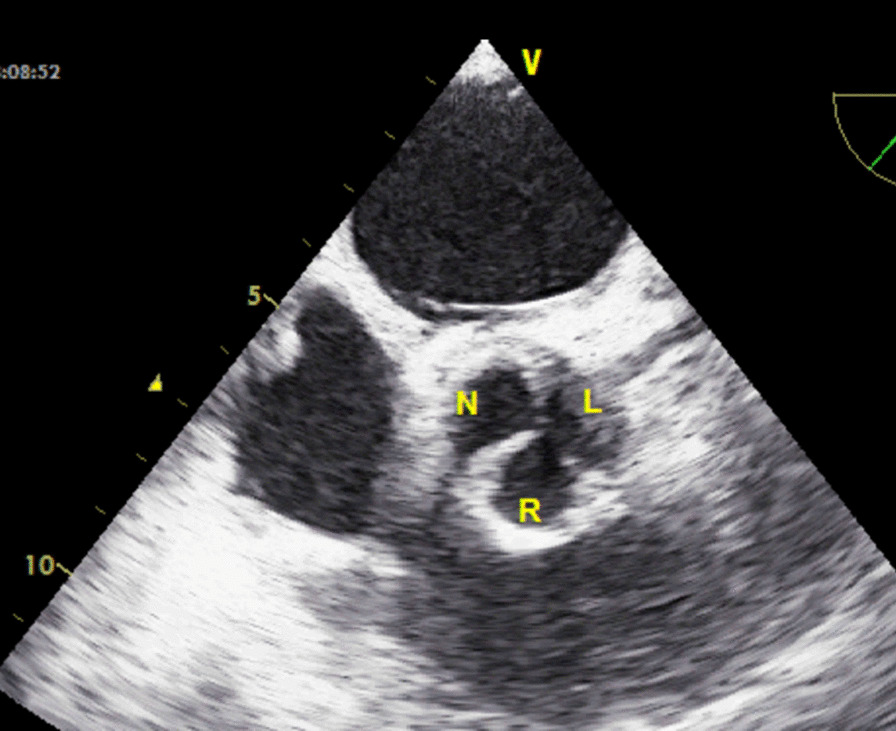
TEE in short-axis view shows good coaptation with no leak

A table for intraoperative use and templates with different aortic annular sizes were constructed (Table 1). Cusp leaflet height was equal to 80% of coaptating margin length. In the operating room, the size of the pericardial leaflet was measured and calculated on the basis of the usual standard diameter of aortic valve sizer. The coaptation of the new aortic valve was evaluated by putting a suction in the left ventricle and creating a negative pressure. Try to design a leaflet and esophageal echocardiography (TEE) also showed 9 mmHg transvalvar gradients and no regurgitation leak (Fig. 3).

**Table 1:** Important parameters for reconstruction of glutaraldehyde treated autologous pericardial valve

Aortic diameter (mm)	Inter commissural distance (mm)	Coapting margin (mm)	Height (mm)	Radius (mm)
19	16.5	30	23	16.5
21	18	32	25	18.5
23	20	36	27.5	20.5
25	22	39	30	22.5
27	23.5	42	32.5	24.5

### Surgical technique

The surgical procedure was performed through a midline sternotomy and pericardiectomy. A large piece of pericardium (10 cm × 10 cm) was harvested and treated with 6.25% glutaraldehyde for 15 min, then rinsed in normal saline for 10 min.

Cardiopulmonary bypass was instituted with aortic and right atrial cannulation. The ascending aorta was cross-clamped, and a transverse aortotomy was performed 1.5 cm distal to the sinotubular junction. Cardioplegia was directly infused into the coronary ostia. The rheumatic aortic leaflets were resected, and the aortic diameter was measured using a standard aortic valve sizer.

In this new formula, acquired parameters for reconstruction of the new aortic cusp can easily be measured for each aortic valve size. If we consider the aortic valve ring as a circle, each leaflet free-edge forms three legs of an equilateral triangle. Based on geometric calculation, the length of each lateral is equal to our formula. The annular margin of the newly tailored cusp is sutured by 4–0 Prolene to the native aortic annulus’s nadir and continues up to the commissural tip.

The Sabzi formula is the calculation of intercommissural distance (ICD).
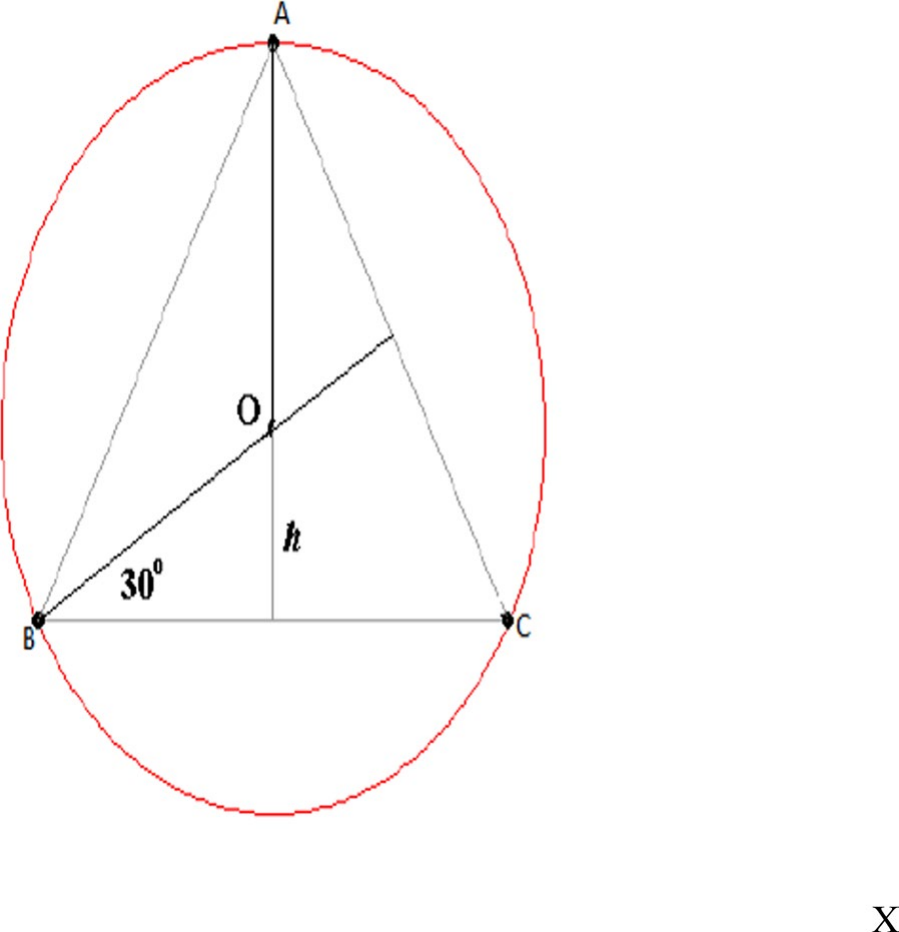

$$B_{1} = 30^\circ \to COSB_{1} = \frac{BH}{{OB}} \to \frac{\sqrt 3 }{2} = \frac{BH}{\frac{X}{2}} \to BH = \frac{\sqrt 3 }{2} \times x$$

$$\text{Inter commissural distance}=BC=2BH\to BC=2\left(\frac{\sqrt{3}}{4}X\right)= \frac{\sqrt{3}}{2}X$$

$$\text{ICD }=\frac{\sqrt{3}}{2}X\times 1.8 =(coaptating\, margin)CM$$

Radius: ICD.

*H* = 80% (coaptating margin) CM.

**Fig. 4:**
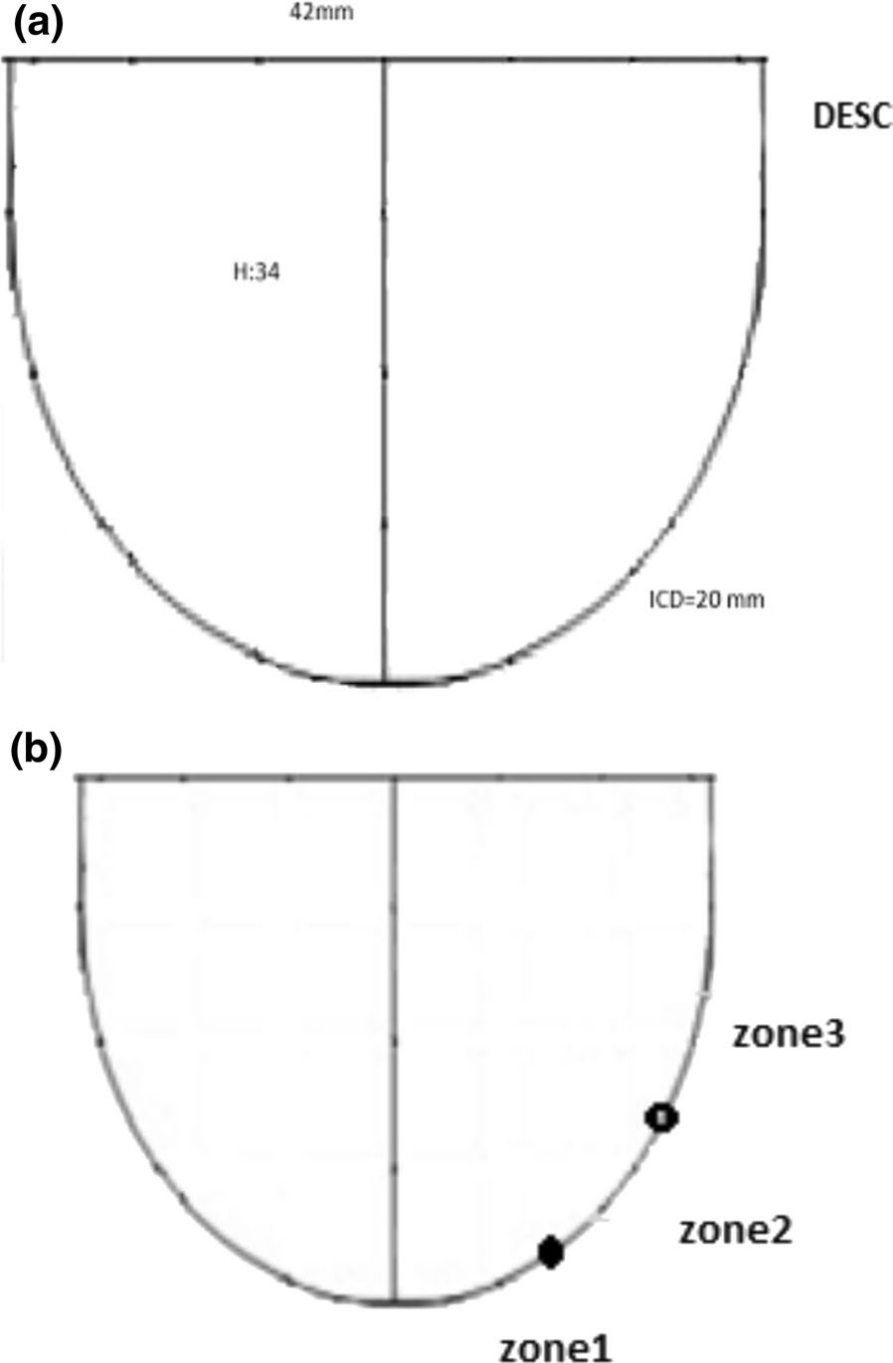
**A** The main parameters for tailoring new cusp are shown. DESC, distal end of semicircle; ICD, intercommissural distance; H, height. **B** The classification of native aortic annulus to three zones for different style of sewing new cusp is shown

With usage of these parameters, new aortic cusp could be measured and tailored in a proper size. The basic parameters of the new cusp are measured by the intercommissural distance, which is computed by using the aortic annulus diameter (D) as a point of entry to the calculation’s sequence (Fig. 4A). The annular margin of the newly tailored cusp is sutured by 4–0 Prolene to the native aortic annulus’s nadir and continues up to the commissural tip. The distance between the nadir and commissural tops is divided into three zones in the annulus (Fig. 4B). At zone 1, the ratio of interval between each suture bite on the new pericardial cusp and aortic annulus is 3.1. This ratio would be reduced to 2.1 at zone 2 and finally declined to 1.1 in the commissural part or zone 3. At the commissural top, two ends of the needle were brought beyond the aortic wall and knotted on the pericardial pledget. For better alignment and proper coaptation, two nearby leaflets in the commissural end were sutured together with a simple u shape 6–0 Prolene suture. Finally, any redundancy of the leaflet was trimmed or reduced by plication with a 6–0 Prolene suture to equalize proper length and height. In the end, the coaptation of the valve was evaluated visually in terms of effective height and good vertical shape similar to a standard three-cuspid native valve and also by putting a suction in the left ventricle to create a negative pressure.

### Postoperative outcome

The aortotomy was closed, and the patient was weaned from cardiopulmonary bypass. Intraoperative TEE showed a well-opening valve with a trivial residual leak and a transvalvular gradient of 9 mmHg. At 3-year postoperative follow-up, the TEE revealed good valve movement and a trivial leak. The patient was in satisfactory condition and had returned to working in a grocery shop and taking daily aspirin (80 mg).

## Discussion

The current study presents a novel technique for aortic valve reconstruction using GTAP, which offers several advantages compared with other existing approaches. One key advantage of the GTAP technique is the avoidance of anticoagulation requirements, as the autologous pericardial leaflets do not require a prosthetic valve [7, 8]. This can improve quality of life for patients, especially younger individuals, who would otherwise need lifelong anticoagulation therapy with mechanical valve replacements.

Additionally, the GTAP approach has demonstrated good durability and resistance to calcification, retraction, and infection—all issues that plagued earlier attempts at using untreated autologous pericardium for aortic valve reconstruction [7, 9]. The glutaraldehyde treatment helps preserve the structural integrity of the pericardial tissue, as evidenced by the excellent valve function observed during long-term follow-up in Ozaki’s report [7]. Before Ozaki, Bjork used autologous pericardium in the repair or replacement of the aortic valve [10]. However, with the untanned pericardium, calcification, shriveling, and scarring were common, and the procedure was abandoned.

Two decades later, Love tanned the pericardium in 0.6% glutaraldehyde and solved the scarring issue [10]. The excellent outcome of GTAP was observed in Al Halees *et al*.’s report [11]. According to Ozaki and colleagues’ report, excellent pericardial valve function was observed after 6 years of follow-up [12]. The annual valve-related complication in Ozaki’s report was 1% with a low rate of reoperation [12]. Compared with transcatheter aortic valve replacement (TAVR), which has emerged as a less invasive option, the GTAP technique still requires open-heart surgery [7]. However, the authors note that GTAP can be applied to a wider spectrum of aortic valve pathologies, including complex cases that may not be suitable for TAVR [7]. Furthermore, the GTAP approach allows for precise tailoring of the pericardial leaflets to the patient’s anatomy, which may improve long-term durability and hemodynamic performance. Potential limitations and challenges of the GTAP technique include the risk of late annular dilatation and other technical failures [8]. Careful patient selection, meticulous surgical technique, and close long-term monitoring will be crucial to mitigate these risks. Additionally, the GTAP procedure requires specialized expertise and may not be widely available, limiting its broader adoption. Overall, the GTAP technique for aortic valve reconstruction appears to be a promising alternative to traditional valve replacement, offering the potential for improved outcomes and quality of life for patients with aortic valve disease. However, continued research and long-term follow-up will be necessary to fully establish its efficacy and safety compared to other surgical and transcatheter approaches.

## Conclusion

The reported surgical technique offers a promising approach for aortic valve reconstruction using autologous pericardium. The detailed preoperative planning and the systematic surgical steps described in this article can potentially improve the outcomes of aortic valve repair procedures, especially in patients with complex aortic valve pathologies. This technique may provide a viable alternative to conventional aortic valve replacement, preserving the patient’s native valve structure and potentially reducing the need for lifelong anticoagulation. Further clinical studies are warranted to evaluate the long-term durability and efficacy of this technique.

## Data Availability

All participants involved in this study have provided explicit consent for the publication of the findings.

## References

[1] Malaisrie SC, McCarthy PM, Otto CM, Bonow RO (2021). Surgical approach to diseases of the aortic valve and aortic root. Valvular heart disease: a companion to Braunwald’s heart disease.

[2] Spadaccio C, Alkhamees K, Al-Attar N (2019). Recent advances in aortic valve replacement. F1000Research.

[3] Brinkman WT, Williams WH, Guyton RA, Jones EL, Craver JM (2002). Valve replacement in patients on chronic renal dialysis: implications for valve prosthesis selection. Ann Thorac Surg.

[4] Ozaki S, Kawase I, Yamashita H, Uchida S, Nozawa Y, Takatoh M (2014). A total of 404 cases of aortic valve reconstruction with glutaraldehyde-treated autologous pericardium. J Thorac Cardiovasc Surg.

[5] Ozaki S, Kawase I, Yamashita H, Uchida S, Nozawa Y, Matsuyama T (2011). Aortic valve reconstruction using self-developed aortic valve plasty system in aortic valve disease. Interact Cardiovasc Thorac Surg.

[6] Aluru JS, Barsouk A, Saginala K, Rawla P, Barsouk A (2022). Valvular Heart Disease Epidemiology. Med Sci (Basel).

[7] Sá MPBO, Perazzo ÁM, Zhigalov K, Komarov R, Kadyraliev B, Enginoev S (2019). Aortic valve neocuspidization with glutaraldehyde-treated autologous pericardium (Ozaki Procedure)—a promising surgical technique. Braz J Cardiovasc Surg.

[8] Albertini A, Raviola E, Zucchetta F, Brega C, Mikus E, Tripodi A (2022). Aortic valve neocuspidization with glutaraldehyde-treated autologous pericardium to avoid the prosthesis-patient mismatch of a severely obese 57-year-old patient—a case report. J Vis Surg.

[9] Kawase I, Ozaki S, Yamashita H, Uchida S, Nozawa Y, Matsuyama T (2013). Aortic valve reconstruction with autologous pericardium for dialysis patients. Interact Cardiovasc Thorac Surg.

[10] Björk VO, Hultquist G (1964). Teflon and pericardial aortic valve prostheses. J Thorac Cardiovasc Surg.

[11] Al Halees Z, Al Shahid M, Al Sanei A, Sallehuddin A, Duran C (2005). Up to 16 years follow-up of aortic valve reconstruction with pericardium: a stentless readily available cheap valve?. Eur J Cardiothorac Surg.

[12] Ozaki S, Kawase I, Yamashita H, Uchida S, Takatoh M, Hagiwara S (2015). Aortic valve reconstruction using autologous pericardium for aortic stenosis. Circulation.

